# UAV Flight and Landing Guidance System for Emergency Situations †

**DOI:** 10.3390/s19204468

**Published:** 2019-10-15

**Authors:** Joon Yeop Lee, Albert Y. Chung, Hooyeop Shim, Changhwan Joe, Seongjoon Park, Hwangnam Kim

**Affiliations:** School of Electrical Engineering, Korea University, Seoul 136-713, Korea; charon7@korea.ac.kr (J.Y.L.); aychung@korea.ac.kr (A.Y.C.); hooyp@korea.ac.kr (H.S.); chjoe01@korea.ac.kr (C.J.); psj900918@korea.ac.kr (S.P.);

**Keywords:** UAV, laser guidance, emergency landing, particle filter, optical flow

## Abstract

Unmanned aerial vehicles (UAVs) with high mobility can perform various roles such as delivering goods, collecting information, recording videos and more. However, there are many elements in the city that disturb the flight of the UAVs, such as various obstacles and urban canyons which can cause a multi-path effect of GPS signals, which degrades the accuracy of GPS-based localization. In order to empower the safety of the UAVs flying in urban areas, UAVs should be guided to a safe area even in a GPS-denied or network-disconnected environment. Also, UAVs must be able to avoid obstacles while landing in an urban area. For this purpose, we present the UAV detour system for operating UAV in an urban area. The UAV detour system includes a highly reliable laser guidance system to guide the UAVs to a point where they can land, and optical flow magnitude map to avoid obstacles for a safe landing.

## 1. Introduction

The unmanned aerial vehicle (UAV) has a wide operating radius and can be equipped with diverse sensing and actuation devices [1]. Through these advantages, UAVs can perform various roles and missions. Recently, a variety of studies have been carried out to apply UAV to real life, such as collecting vehicle traffic information [2,3], surveillance systems for cities [4,5,6], constructing network infrastructure [7,8], and delivering products [9]. In particular, many companies are investing in the field of delivery systems using UAV. Amazon Prime Air patented a UAV delivery system [10]; DHL tested its parcel copter in Germany; and various delivery companies such as UPS, USPS, Swiss Post, SF Express and Ali Baba started researches related to UAV delivery system. These delivery systems often deliver small items ordered by people, so UAVs often operate in densely populated cities. This is possible because UAV can move freely in three dimensions, so it can deliver goods to destinations in high-rise buildings or apartments.

However, the city has many elements that interfere with the UAVs’ flight. Buildings make it difficult to obtain a line of control (LOC) for UAV control and degrade the accuracy of GPS-based positioning. There are studies that the use of UAV is difficult due to the low stability of GPS in urban areas [11,12]. Without GPS, the autonomous waypoint flight of the UAV is not possible. Also, if wind drift occurs, UAV will not be able to calibrate its position. UAV can hardly plan its flight for missions when GPS service is degraded. In addition, RF interference and multi-path effects can interfere with the flight of UAV in the urban area [13]. In this case, the signal for manipulating the UAV is disturbed, so that the operator cannot maneuver the UAV remotely. UAVs that are thus disturbed during their flight are more likely to cause accidents in urban areas. Therefore, we need a flight and landing guidance system for public safety against UAVs that lose connection and position (and even malfunctioned UAVs).

For these purposes, we propose the UAV detour system that satisfies the following conditions.

- UAV should be allowed to continue its mission in situations where GPS is not available or the network is disconnected;
- The system that guides the UAVs’ flight should be able to overcome multi-path fading and interference that may occur in urban areas;
- When the UAV is landing, the UAV must be able to land safely while avoiding obstacles.

The UAV detour system that satisfies the aforementioned conditions has two subsystems. The first is the flight guidance system, and the second is the safe landing system. The flight guidance system can guide UAV to desired landing points using laser devices that are free of multi-path effects and other interference common in radio waves. When a UAV with flight guidance system detects a laser, the UAV moves in the direction of the incoming laser. Also, the flight guidance system is based on a particle filter. The other system, safe landing system provides obstacle avoidance for UAV. The safe landing system was developed based on our previous work [14]. In addition, it has been improved for the use of optical flow magnitude maps, which make it more stable on a low-power computing board in UAV than in previous work. When the UAV is flying or landing, the UAV can extract the optical flow from the images taken by the mounted camera. The safe landing system analyzes the optical flow magnitude map to identify obstacles and maneuver the UAV away from obstacles. Overall, the safe landing system allows UAV to safely land without collisions even when GPS or network assistance is unstable. The UAV detour system was installed on a real UAV and tested in an actual environment to see if it could steer UAV and land it safely in urban area [15,16,17].

The rest of the paper is organized as follows. Section 2 reviews related work and background theory. Detailed explanation of the UAV detour system is described in Section 3. Section 4 describes the detailed implementation of the UAV detour system, and Section 5 presents the experiments and evaluation of the proposed system. Section 6 describes the limitations of UAV detour system and plans to improve it. Finally, Section 7 concludes the paper.

## 2. Preliminaries

### 2.1. Related Work

There have been a number of previous studies related to the unmanned aerial vehicle (UAV) detour system. Descriptions of related techniques are described below.

#### 2.1.1. Laser Guidance Systems

Lasers have the advantage of being able to distinguish easily from other light sources. Focusing on the advantages of lasers, there have been studies to use lasers in the guidance of UAV.

Vadim et al. proposed a system for delivering GPS-based flight information to the UAVs via a laser [18]. The information transmitted through the laser includes both flight path and landing location information. However, the authors did not implement the system on real UAVs. The system proposed by Shaqura et al. aims to detect the laser point by applying image processing on images taken by the camera mounted on the UAV. The laser guides UAV to landing point [19]. However, since these works provide flight information based on the UAVs’ GPS, it is difficult to expect successful operation in an environment where the GPS signal is degraded due to the environment.

In an emergency situation, there was an attempt to guide a UAV based on a laser without relying on GPS. The system proposed by Jang et al. used a laser to hold the current position of UAVs in case of deteriorated GPS signal [20]. This paper assumes that the UAVs are flying in an electric wave-shaded region, preventing the drift of the UAVs and holding the position through a number of photo-resistors. The system presented in this paper does not guide over long-distance, but shows that flight stability can be improved by using laser even in the environment without GPS.

Most of the proposed laser guidance systems were GPS dependent or had a short guidance range. However, our proposed flight guidance system was designed to take advantage of the long-range of lasers, assuming flight in a GPS-denied environment. The proposed system can reliably recognize the laser and determine the flight direction through the particle filter, which has been modified to be suitable for the real UAV flight. Particle filter had proven to be useful in state estimation problems such as simultaneous localization and mapping (SLAM) of robot research area [21]. In addition, Hightower et al. implemented a multi-sensor localization system based on particle filter and presented performance comparison showing that it is practical to run a particle filter on handheld devices [22]. Research on particle filters has continued over the last few decades and has been applied to address non-Gaussian distributions in various fields.

#### 2.1.2. Obstacle Avoidance Studies

In order for a UAV to fly or land in an urban area, the UAVs must be able to avoid obstacles. There have been studies to implement obstacle avoidance based on optical flow. Lorenzo et al. proposed an optical flow-based landing system [23]. However, the system proved its performance only by simulation. Souhila et al. applied obstacle avoidance based on optical flow to robots [24]. The algorithm proposed in this study determines that there is an obstacle if an optical flow value imbalance is detected while the robot is moving. Similarly, Yoo et al. applied an algorithm to the UAV navigation system to avoid obstacles based on the imbalance of optical flow values [25]. However, these studies had limited movement because they could only maneuver the robot or UAV in the left and right directions. Our proposed safe landing system can avoid the obstacles in all directions based on optical flow, and can effectively cope with the complex environment and various obstacles in the urban area. Miller et al. estimated reliable altitude using the difference between the optical flow velocity and calculated via exact formulas [26]. Estimation of altitude is tested from video sequences obtained in flights, but altitude is not calculated during the actual flight. Herisse et al. presented a nonlinear controller for the vertical landing of a VTOL UAV using the measurement of average optical flow with the IMU data [27]. Since VTOL is assumed to be equipped with a camera and IMU, there is a difference between our research in the case of using only cameras.

In addition, there have been studies to avoid obstacles with various techniques. Mori et al. showed a technique for determining obstacles, using the SURF [28]. In the paper, the authors proposed a system that determines obstacles when a large image difference is detected by comparing each image frame by frame. The author-proposed system used the phenomenon in which objects nearer to the camera become larger as the camera moves towards the objects. Hrabar et al. used stereo vision to identify obstacles [29]. The authors created a 3D map of the surrounding terrain using the images shown around them. This assisted UAVs to avoid obstacles. Ferrick et al. used LIDAR [30]. LIDAR utilizes laser to estimate the distance to nearby objects in real-time and detect the approaching object. In addition, as shown in Kendoul’s survey paper [31], there are various techniques that allow UAVs to avoid obstacles through autopilot in the event of GPS or communication failures.

#### 2.1.3. Autonomous UAV Landing Systems

There have also been studies that use image processing for autonomous landing. In order for the UAV to recognize the landing point, several studies have proposed to use landing markers at landing points.

Bi et al. proposed a system for calculating the relative position of a UAV with a marker and landing a UAV toward the marker’s position [32]. Lange et al. proposed a system that controls the speed of the UAVs by estimating the relative altitude and position of the marker [33]. Venugopalan et al. placed a landing marker on the autonomous marine vehicle to land the UAVs on it [34].

Using the marker makes it easy for the UAVs to identify the landing point and allows more precise landing. In order to land the UAV in an area where the markers are not ready, some of the studies have used optical flow to identify the landing area. Cesetti et al. proposed a system for identifying safe landing sites using optical flow [35]. In this paper, the depth map of the ground is drawn using optical flow and feature matching. Then, Cesetti et al. analyzed the flatness of the depth map to determine the safe landing area. Similar to [35], Edenbak et al. proposed a system for identifying safe landing points [36]. This system identifies the structure of the ground via optical flow.

Our proposed UAV detour system does not require any special markers to land, and automatically determine the flat ground that can be landed. In addition, the proposed system includes the flight guidance system that can guide UAVs to landing points even over long distances. The proposed system was modified to be suitable for real UAV and proved its performance through actual experiments.

### 2.2. Background

This section briefly introduces the particle filter, the core theory of our proposed flight guidance system, and the optical flow method used in the safe landing systems.

#### 2.2.1. Particle Filter Theory

The sequential Monte Carlo method, also known as particle filtering or bootstrap filtering, is a technique for implementing a recursive Bayesian filter by Monte Carlo simulations. The key idea is to represent the required posterior density function by a set of random samples with associated weights and to compute estimates based on these samples and weights. As the number of samples becomes very large, this Monte Carlo characterization becomes an equivalent representation to the usual functional description of the posterior pdf [37].

#### 2.2.2. Optical Flow Method

Optical flow is defined as the pattern of apparent motion of objects in a visual scene caused by the relative motion between an observer and a scene. In addition, Lucas–Kanade algorithm is one of the simple optical flow techniques which can provide an estimate of the movement of interesting features in successive images of a scene. Lucas–Kanade algorithm bases on two assumptions that two images between frames are separated by a small time increment and movement of objects is not significant. Moreover that the spatially adjacent pixels tend to belong in same object and have identical movements, constant brightness [38].

## 3. Methods

### 3.1. System Overview

**Figure 1:**
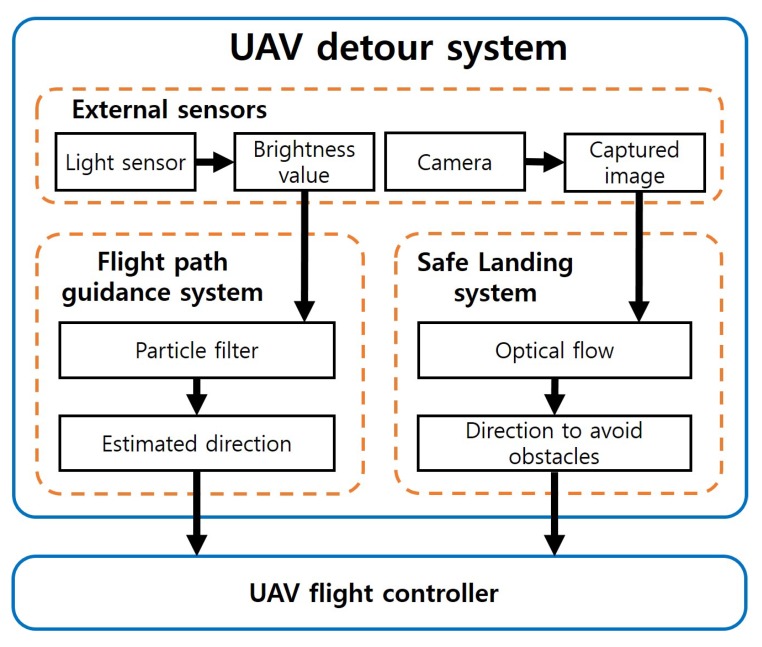
Overview of the unmanned aerial vehicle (UAV) detour system.

As shown in Figure 1, the UAV detour system has two major subsystems. One of the subsystems is the flight guidance system, that uses the laser to maneuver the UAV in the desired direction. The flight guidance system detects the laser with a light sensor mounted on the UAV. However, the laser that is detected by the light sensor is not linear. To reliably detect the laser and determine the direction the UAV will fly, the flight guidance system uses particle filter theory to estimate the direction based on the direction the light is coming from.

The other subsystem is the safe landing system that can identify obstacles on the landing area with an optical flow magnitude map. The safe landing system obtains images through a camera mounted on the UAV. By analyzing the image, optical flow information can be obtained. The safe landing system analyzes the magnitude of the obtained optical flow to determine the obstacles below the UAV. When the UAV determines that there are obstacles at the landing area, the UAV lands with avoiding obstacles. After operations of subsystems, the maneuver commands of the UAV generated from the two subsystems are transferred to the UAV controller so that the UAV detour system can be applied to the movement of the actual UAV.

### 3.2. Flight Guidance System for UAV

In order to increase the accuracy of flight direction through laser detection, a modified particle filter was used in the flight guidance system. A variety of adjustments have been applied to the modified particle filter, considering that it operates on a UAV.

#### 3.2.1. Particle Filter Based Flight Guidance

In the flight guidance system, when the measured brightness value exceeds the threshold at multiple sensors, the sensor with the brightest light (laser) is identified and finally controls the next flight direction of the UAV. If the UAV detects the incoming laser, UAV computes the bearing angle of the laser and moves at a given constant speed to the detected direction. However, moving the UAV and detecting the direction of the laser during moving cannot be linearly described, we used particle filter to improve the accuracy of laser identification. The particle filter recursively estimates the sequence of system states (approximated direction to the source of the light) from the sensor measurements. Filtering via sequential importance sampling (SIS) consists of recursive propagation of importance weights $w_{k}^{i}$ and support points $l_{k}^{i}$ as each measurement is received sequentially [37].

**Figure 2:**
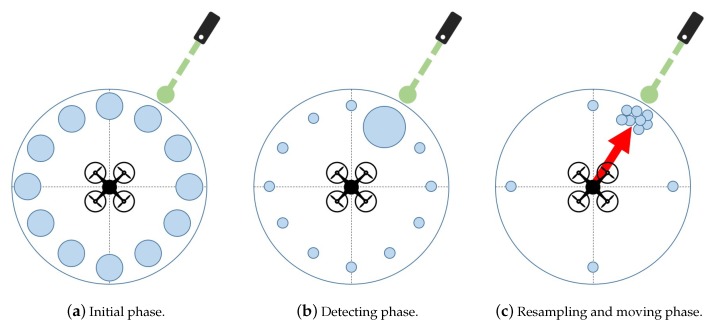
Examples of particle-applied flight guidance algorithm.

Figure 2 shows a brief overview of the particle-applied flight guidance system. The blue circles represent the weight of each particle. As shown in Figure 2a, the particles are uniformly distributed in all directions. Then, as shown in Figure 2b, the weight of the particle in the direction in which the laser is detected is getting larger. As can be seen in Figure 2c, more particles are placed where there is a larger weight value. At this time, the flight guidance system prepare for situations in which the direction of the light changes by placing a small number of particles on the sensors in different directions. Finally, the UAV can fly to the direction of the most particles.

To guide the UAV exactly in the intended direction, they must maintain information about the direction along which the laser will guide. So, the target direction can be described by the state vector $l_{k}$ as follows:

$$l_{k}={\left[x_{k},y_{k},\theta_{k}\right]}^{T},$$

where *k* is the discrete-time, *T* denote transpose, $x_{k}$ and $y_{k}$ denote Cartesian coordinates at time=k which can be calculated from the bearing angle $\theta_{k}$ pointing to the laser light source.

In order to successfully guide the UAV using laser light, the operator must continually irradiate the laser in one direction unless an exceptional situation occurs. It can be expected that the previous state values will be changed slightly. Thus, the state transition equation for the flight guidance system can be written as

$$l_{\left(k+1\right)}=F_{k}l_{k}+v_{k},$$

where $v_{k}$ is the white Gaussian process noise and $F_{k}$ is the state transition matrix can be written as

$$F_{k}=\left[\begin{array}{ccc} \frac{cos(\pi /2-\left(\theta_{k}+v_{k}\right))}{x_{k}} & 0 & 0\\ 0 & \frac{sin(\pi /2-\left(\theta_{k}+v_{k}\right))}{y_{k}} & 0\\ 0 & 0 & \frac{\theta_{k}+v_{k}}{\theta_{k}} \end{array}\right].$$

Based on this matrix, we update the current state’s bearing angle $\theta_{k}$ by adding the process noise $v_{k}$ to the current value and then calculate the next state’s coordinates $x_{\left(k+1\right)}$ and $y_{\left(k+1\right)}$. On the one hand, the measurement equation can also be defined as

$$z_{k}=h\left(l_{k}\right)+e_{k},$$

where $z_{k}$ is the measurement and $e_{k}$ is the white Gaussian measurement noise. The nonlinear measurement function $h(l_{k})$ can be denoted as

$$h\left(l_{k}\right)=\theta_{k}=tan^{-1}\frac{y_{k}}{x_{k}},$$

when sensors detect the light incidence, they convert the direction of the light into polar coordinates in two dimension. Therefore, we define $h(l_{k})$ as a part of measurement equations with formulas changing Cartesian coordinates into polar coordinates and assume that the radius is always 1 when transforming the coordinate system. Note that the flight guidance system was developed to maneuver UAV in two dimensions but it can be extended to maneuver UAV in three dimensions for UAV flight on building rooftops or in complex terrain.

#### 3.2.2. Resampling Method of Particle Filter

There are numerous versions of the resampling method in the field of a particle filter. Thus, it is important to choose an efficient method because each resampling method has different complexity depending on the operational algorithm. Considering the simplicity of implementation and the efficiency of the algorithm, systematic resampling was applied to the flight guidance system [37]. The most serious problem is that some particles with large weights are inevitably selected during the resampling and sample impoverishment occurs as the diversity of the sample decreases. As mentioned earlier, the flight guidance system must accurately estimate the direction that the UAV should travel. Therefore, under the assumption that the incoming direction of light is constant, it may be helpful to have less sample diversity. However, if the direction of the light guiding the UAV is suddenly changed, the exact direction cannot be estimated due to the effect of the sample impoverishment which becomes too severe. To solve sample impoverishment, the flight guidance system maintains a minimum level of sample diversity and combines the sample dispersion process with the resampling method. The sample dispersion allows a given portion of the total number of samples to be redistributed evenly over the entire state space. In the resampling algorithm, to determine how severe the weight degeneracy is before conducting the resampling process, it estimates an effective sample size ${\hat{N}}_{\mathrm{eff}}$ as follows

$${\hat{N}}_{\mathrm{eff}}=\frac{1}{\sum_{i=1}^{N_{s}}{\left(w_{k}^{i}\right)}^{2}},$$

where $w_{k}^{i}$ is the normalized weight, $N_{s}$ is the number of particles. After a certain number of recursion steps, all but one particle have negligible normalized weights, which is called weight degeneracy. As the degeneracy phenomenon becomes more severe, the ${\hat{N}}_{\mathrm{eff}}$ value approaches zero. Therefore, if ${\hat{N}}_{\mathrm{eff}}$ is smaller than the predefined threshold $N_{T}$ (e.g., $N_{T}=N_{s}/2$), the resampling process is executed to mitigate the degeneracy phenomenon. Here, lowering the $N_{T}$ value solves the weight degeneracy problem, but the resampling process is frequently performed, resulting in system performance problems. On the contrary, if the value of $N_{T}$ is higher than $N_{s}/2$, the opposite situation occurs and it can be regarded as a trade-off relationship. In addition, after setting all weight values to $1/N_{s}$ during the resampling process, the sample dispersion is executed to avoid the problem of sample impoverishment. In the flight guidance system, only 10% of the total number of samples are uniformly dispersed across the state space by selecting the bearing angle $\theta_{k}$ of each state vector within the range of direction values in which light can be irradiated.

#### 3.2.3. Delay Reduction Analysis through Sample Dispersion Modeling

**Figure 3:**
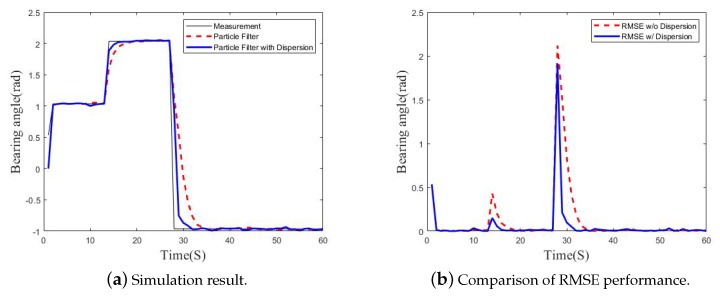
Performance of particle filter with sample dispersion.

The sample dispersion method was applied to the particle filter of the flight guidance system so that the UAV can respond to incoming laser in various directions. Figure 3a shows simulation result of particle filter with sample dispersion. Assuming that the incident angle of the laser light varies suddenly about 180 degrees, the particle filter using the sample dispersion accurately estimated the value close to the measurement almost twice as fast as the particle filter without the sample dispersion. Figure 3b shows root mean square error (RMSE) performance of our simulation result. A large error values are shown due to a sudden change of the incident angle, but a particle filter with sample dispersion minimizes error about twice as fast as the general particle filter.

#### 3.2.4. Optimal Number of Particles through Modeling

Determining the statistically efficient number of samples is very important in the particle filter, as it is possible to estimate the expected value more accurately as the number of particles increases, but at the same time the computational complexity also increases. So we simulated the number of particles suitable for calculating the direction of the UAV flight and the results are shown in Table 1. Through this simulation, we can obtain the error between the constant measurements and the estimations from the particle filters with a different number of particles. When the number of particles is 500 or greater, the error with the measurements is less than 1 degree.

In the flight guidance system, the direction of flight can be accurately calculated even with a small number of particles. In addition, considering that UAVs run the proposed system based on single-board computer with a low processing capacity using Lithium-ion batteries, the number of particles suitable for the flight guidance system is 500.

### 3.3. Safe Landing System for UAV

In order to allow the UAV to make a safe landing, the safe landing system was able to identify obstacles based on the optical flow and avoid the obstacles.

#### 3.3.1. Optical Flow Based Obstacle Avoidance

**Figure 4:**
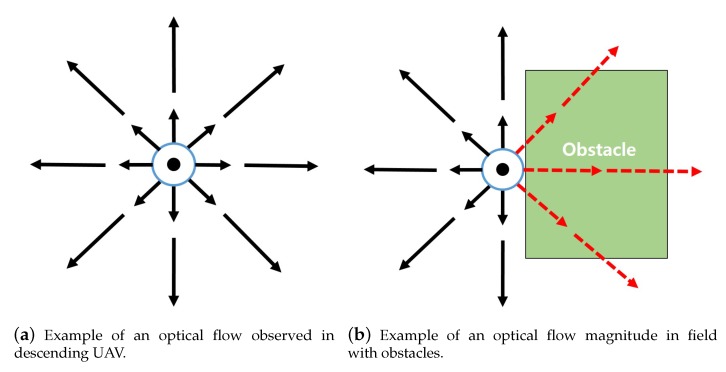
Examples of optical flow magnitude map.

**Figure 5:**
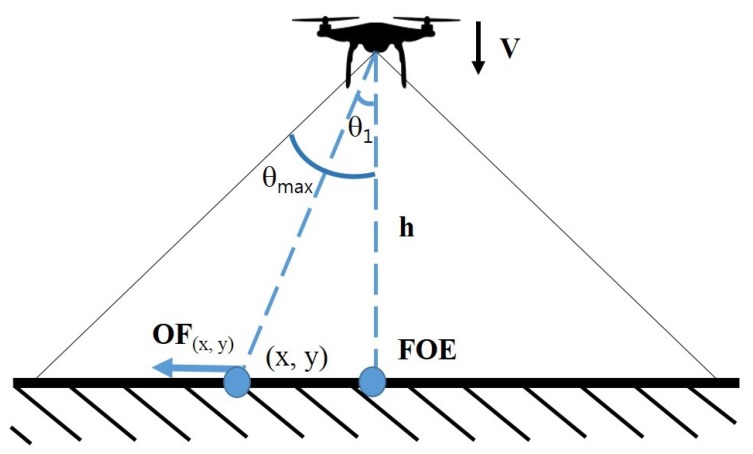
Optical flow of a descending UAV.

When a UAV with a downward facing camera descends, the image sequences captured from the camera produce optical flow that spreads out from a point called focus of expansion (FOE). By locating FOE and analyzing the patterns of the optical flow, optical flow magnitude module calculates optical flow magnitude to estimate the heading of the UAV and the structure of the environment beneath the UAV. Figure 4 illustrates an optical flow map processed from a descending UAV with downward facing camera on a flat surface. Figure 5 illustrates the operation of calculating optical flow. The magnitude of the optical flow can be formulated with the following equation:

(1) $$|{\vec{OF}}_{x,y}\left|\approx \alpha \right|\vec{v}|\tan\theta .$$

In Equation (1), ${\vec{OF}}_{x,y}$ is the optical flow value at (x,y), $\vec{v}$ is the descending speed of the UAV, θ is the angle that between the camera to FOE and camera to point (x,y), and α is the scale factor of the camera. Since θ is greater for the points farther away from FOE, the magnitude of the optical flow for points farther away from FOE is greater than for the points closer to FOE. Furthermore, points with the same distance from FOE will have the same optical flow magnitude.

By analyzing the optical flow magnitude map, the optical flow magnitude module estimates the structure of the ground. If the surface beneath is flat, the optical flow magnitude observed around the FOE is balanced. For surfaces where obstacle exists, the magnitude of optical flow shows deformation at the location where obstacle exist as shown in Figure 4b. Locations where the altitude is higher than the rest, the angular acceleration of θ becomes greater than other locations that lie on the same distance from FOE in the image. The increased angular acceleration of θ results in greater optical flow magnitude and the optical flow magnitude map shows the unbalanced magnitude of optical flows around FOE.

#### 3.3.2. Optical Flow Modeling

The safe landing system system identifies obstacles through the optical flow. However, if the magnitude of optical flow is small, it is difficult for the UAV to detect the obstacle. Because the UAV fly at high altitudes, the variation in optical flow is very small. Therefore, we have confirmed through modeling that the magnitude of optical flow measured in the UAV is large enough to detect obstacles prior to experiments using real UAVs.

To model the optical flow, we assume that the UAV is equipped with a camera with a 2 × $\theta_{\max}$ viewing angle, as shown in Figure 5. When we look at the point at the camera to calculate the optical flow at the angle of $\theta_{1}$, the coordinates $OF_{\left(x,y\right)}$ shown can be expressed by the following equation:

(2) $$OF_{\left(x,y\right)}=h\times \tan\left(\theta_{1}\right).$$

The magnitude of optical flow(${\vec{OF}}_{x,y}$) can be obtained by calculating the point at which the $OF_{\left(x,y\right)}$ point is observed at the image pointing to the camera after the UAV descend to the *d* altitude, and the equation is as follows:

(3) $$\begin{array}{cc} {\vec{OF}}_{x,y} & =\frac{h\times \tan(\theta_{1})}{\left(h-d\right)\times \tan\left(\theta_{\max}\right)}-\frac{h\times \tan(\theta_{1})}{h\times \tan(\theta_{\max})}\\ \; & =\frac{d\times \tan(\theta_{1})}{\left(h-d\right)\times \tan\left(\theta_{\max}\right)}. \end{array}$$

If an obstacle with a height of *g* exists, the magnitude of the optical flow measured at the UAV increases by the height of the obstacle. For example, if there is an obstacle to the right of the camera image, the magnitude of optical flow difference is calculated as follows:

(4) $$\Sigma ∥\vec{W_{L}}∥-\Sigma ∥\vec{W_{R}}∥=\frac{d\times \tan(\theta_{1})}{\left(h-d\right)\times \tan\left(\theta_{\max}\right)}-\frac{d\times \tan(\theta_{1})}{\left(h-g-d\right)\times \tan\left(\theta_{\max}\right)}.$$

**Figure 6:**
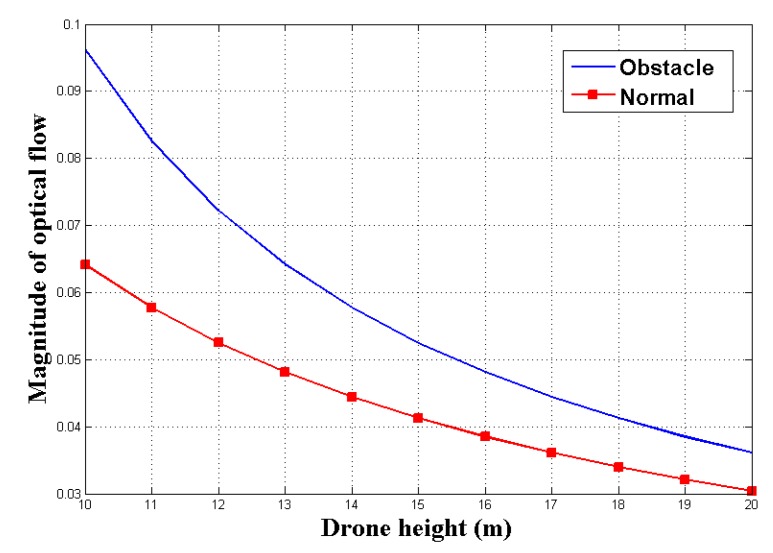
Magnitude of optical flow modeling.

$\Sigma ∥\vec{W}∥$ indicates the magnitude of optical flow in either the left or right region of the camera image. In order to verify the magnitude of optical flow, we set up a simulated flight environment similar to the real UAV experiment. When the UAV equipped with a camera with an angle of view of 90° ($\theta_{\max}$ = 45°) is flying, the angle for determining the optical flow($\theta_{1}$) is set to 30°. When there is an obstacle with a height of 1 m (*g* = 1), the UAV measures the optical flow while lowering the altitude by 1 m (*d* = 1). Figure 6 shows how the magnitude of the optical flow(${\vec{OF}}_{x,y}$) is measured when the UAV is at an altitude of 10 m to 20 m.

As a result of the modeling, when the UAV is flying at a height of 10 m, the magnitude of optical flow is 1.50 times higher than that of the flat surface when the obstacle is present. Also, when the height of the UAV was 20 m, the magnitude of optical flow showed a difference of 1.18 times. These results show that the obstacle can be identified through the magnitude of optical flow at the height at which the UAV is normally flying.

## 4. System Implementation

**Figure 7:**
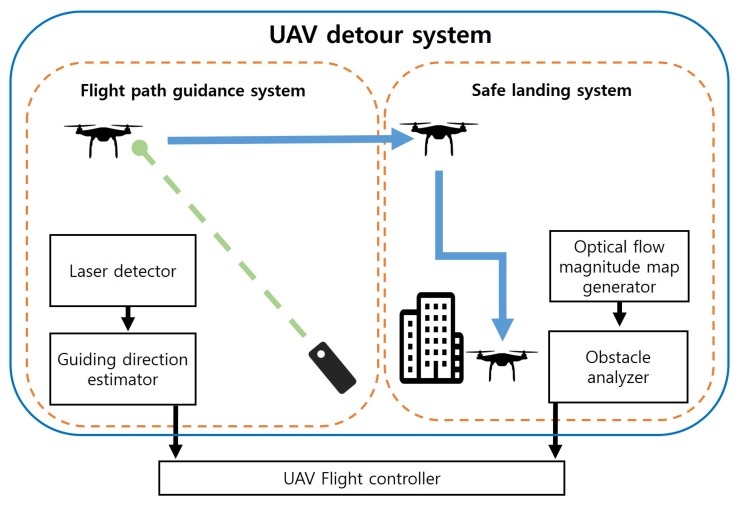
Overall systems and modules.

This section details the algorithms of subsystems and implementation techniques applied to the actual UAV. As shown in Figure 7, UAV detour system consists of two major subsystems.

### 4.1. Flight Guidance System

#### 4.1.1. Laser Detector

The laser detector identifies the incoming direction of the laser through light sensors. The laser detector consists of 12 light sensors arranged in a circular shape, which identifies the incoming direction of the laser. By assigning 30 degrees to each sensor, 12 sensors can cover 360 degrees in all directions. If the higher brightness is measured above a certain threshold than the initial brightness, the light sensor determines that the laser light has been received. In addition, when laser light is detected, the middle value of the range assigned to each sensor is returned.

Furthermore, as the distance increases, the area where light enters becomes larger, so that adjacent sensors can detect light at the same time. Also, the sensors can be affected by the momentary reflection or scattering of other light. In this case, the angular range of each sensor is added up and then the middle value is returned. Then, the laser detector transmits the bearing angle of incoming laser to the guiding direction estimator that returns the direction where the UAV is guided.

#### 4.1.2. Guiding Direction Estimator

The guiding direction estimator operates the particle filtering based on the measurement value received from the laser detector to approximate the direction in which the UAV will be guided. The full algorithm of guiding direction estimator is presented in Algorithm 1.

**Algorithm 1** Guiding direction estimator.
   ***Initialization***
1:discrete-time *k* = 02:**for**i=1, i++, $i==N_{s}$ ($N_{s}$ = number of particles) **do**3:    Initialize_state_vector $l_{0}^{i}$4:**end for**5:**while** measurment $z_{k}=true$ **do** ***Weight update***
6:    **for** i=1, i++, $i==N_{s}$ **do**7:        $l_{k}^{i}∼p\left(l_{k}|l_{k-1}^{i}\right)$8:        ${\tilde{w}}_{k}^{i}∼{\tilde{w}}_{k-1}^{i}p\left(z_{k}|l_{k}^{i}\right)$9:    **end for** ***Normalize***
10:    **for** i=1, i++, $i==N_{s}$ **do**11:        $w_{k}^{i}={\tilde{w}}_{k}^{i}∕\sum_{i=1}^{N_{s}}{\tilde{w}}_{k}^{i}$12:    **end for** ***Resampling based on effective sample size***
13:    ${\hat{N}}_{\mathrm{eff}}=1∕\sum_{i=1}^{N_{s}}{\left(w_{k}^{i}\right)}^{2}$14:    **if** $\;\mathrm{then}\;{\hat{N}}_{\mathrm{eff}}<N_{T}$15:        Resampling ($l_{k}^{i}$)16:        Sample dispersion $\;\theta_{k}^{i}=\theta_{k}^{i}+u\left[-\frac{\pi }{2},\;\frac{\pi }{2}\right]$17:    **end if** ***Flight direction estimation***18:    $S_{k}=\sum_{i=1}^{N_{s}}w_{k}^{i}l_{k}^{i}$19:    $\mathrm{Operate}\_\mathrm{flight}\_\mathrm{controller}\leftarrow \mathrm{Get}\_\mathrm{direction}(S_{n})$20:    k=k+121:**end while**


The Algorithm 1, guiding direction estimator, works in the following steps. First, in the initialization phase, the system initializes the bearing angle $\theta_{k}$ to have different values throughout the entire state space to evenly distribute each particle in all directions. Then, the system checks to see if it has received the measurement from the laser detector because it will start the estimation process with a particle filter after the laser light is detected. Second, the flight guidance system draws the samples from the transitional prior $p(l_{k}|l_{k-1}^{i})$, because we have chosen the proposal distribution $q(l_{k}|l_{k-1}^{i},z_{k})$ as the transitional prior. Third, the selection of the proposal distribution can simplify the weight update equation and update the weight using the likelihood $p(z_{k}|l_{k})$. Fourth, in the normalization step, the weight of each sample (${\tilde{w}}_{k}^{i}$) is divided by the total sum to make the sum of the normalized weights $\sum w_{k}^{i}$ to be 1. Fifth, the system calculates the effective sample size ${\hat{N}}_{\mathrm{eff}}$ and determines that the weight degeneracy problem becomes severe when ${\hat{N}}_{\mathrm{eff}}$ is less than the threshold $N_{T}$. In this case, the system performs the resampling process and applies the sample dispersion method described in Section 3.2.2 to quickly respond to drastic changes in measurements. Finally, the guiding direction estimator obtains an estimated bearing angle ($S_{k}$) and transmits the direction calculated from the estimated bearing angle to the flight controller. Then, the flight controller maneuvers the UAV based on the direction.

### 4.2. Safe Landing System

This subsection describes the optical flow that is the basis of our obstacle avoidance and describes the two modules installed in the safe landing system, the optical flow magnitude map generator and obstacle analyzer.

#### 4.2.1. Optical Flow Magnitude Map Generator

Optical flow magnitude map generator calculates optical flows between frames from the images obtained by the camera. In this process, we considered the situation that the optical flows should be computed on low-power computing boards mounted on UAV. As computing the optical flow for all pixels and drawing a magnitude map [39] has a large load to run on the low-power computing board, it is inappropriate for real-time operation. Therefore, the Lucas–Kanade algorithm [38] could be considered, which sets up a pixel window for each pixel in one frame and finds a match to this window in the next frame for specific pixels extracted with some standards. However, the Lucas–Kanade algorithm has the challenge that it cannot calculate large movement.

Therefore, the optical flow magnitude map generator used the iterative Lucas–Kanade method with pyramids [40], which can supplement this disadvantage. The specific pixels used for the iterative Lucas–Kanade determined by goodFeaturesToTrack function on openCV [41], which detects the strong corner on the image which is easy to trace its movement. Thus, using the benefit of calculating only for certain pixels, not for the entire pixels, the load for computing board can be reduced and does not cause performance degradation on operating.

#### 4.2.2. Obstacle Analyzer

An obstacle analyzer determines the existence of obstacles, and two criteria can be considered. One is the magnitude of optical flow and the second is the feature point, both are from optical flow magnitude map generator. In Section 3.3.2, we modeled magnitude of optical flow. It shows that if an obstacle exists, it has a larger magnitude than the normal, and the closer it is, the larger it becomes. Also, the feature points extracted form goodFeaturesToTrack function are extracted mainly on the obstacles, because obstacles not only have a visual difference in color or pattern against landing point, but also the difference in height against the landing point. In addition, the image in which the obstacle exists creates more feature points than the flat image. Therefore, the greater the number of feature points and the larger the optical flow magnitude, the higher the probability that the obstacle actually exists. In our system, we used the metric to multiply the optical flow by the feature point and use it to identify obstacles. Using this metric, the location of the obstacle can be determined depends on where it is located in the image obtained through the camera. The image is divided into m × m arrays of segments, creating a total of m${}^{2}$ segments per image. The value of *m* can be freely selected according to the experimental situation, such as 3, 5, and 7. In evaluation, *m* is set to 3 and the image is divided into 3 × 3, nine segments. As the obstacles that UAVs face in urban canyons would be large in size (e.g., trees and buildings), setting *m* to 3 is considered to be sufficient to identify the location of the obstacle and avoid it. The value of the metric is derived significantly from the segmented screen where the obstacle is located, and the UAV recognizes that the location obstacle exists, and then flies to the opposite direction.

Algorithm 2 presents the algorithm of obstacle analyzer. When the frames come in through the camera, the obstacle analyzer uses the extracted feature points and calculated magnitude of optical flows from the optical flow magnitude map generator. First, the obstacle analyzer divides the location of feature points in several directions according to the coordinates of the feature points. The directions can be multiple directions, and in Algorithm 2, the directions are set to eight directions. Second, the obstacle analyzer adds the magnitude of optical flow at the feature point to the direction, and repeats this process on every point. By this method, the optical flow for a particular direction becomes proportional to the magnitude and the number of feature points, and if the value is greater than the empirical static threshold value $O_{T}$, it is determined that an obstacle exists in a particular direction.

**Algorithm 2** Obstacle analyzer.

1:
**while** optical_flow_exists==true **do**
2:    **for** i=0, i++, i<number_of_directions **do**3:        **if** location_of_feature_point(x,y)==DIRECTION(i) **then**4:             optical_flow_DIRECTION(i)+=magnitude_optical_flow(x,y)5:        **end if**6:        **if** $optical\_flow\_DIRECTION\left(i\right)>O_{T}$ & $Variance>V_{T}$ **then**7:             exist_of_obstacle_DIRECTION(i)=true8:        **end if**9:    **end for**10:
**end while**




During the process, the magnitude of optical flow in the segmented screen can be measured evenly large when the camera is facing the ground without obstacle after avoiding it. The first reason for this case is because the strong corners that affect the goodFeaturesToTrack function are even on every obstacle-free ground, and the second is because of the tendency that the magnitude of optical flow measured at each segment screen can be similar on the flat ground. For these reasons, if only the magnitude of optical flow is used, unintentional situations where the obstacle analyzer misunderstands a flat ground as an obstacle can occur. In order to prevent this case, the obstacle analyzer additionally utilized another metric, the variance of optical flow magnitude values measured at each segment screen. When analyzing the optical flow map in an obstacle environment, the difference between the optical flow magnitude values measured on the segment screen with obstacles and the segment screen without obstacles is huge. Therefore, in the obstacle environment, the variance of optical flow magnitude values increases. Thus, the obstacle analyzer only performs detection of obstacle in situations that variance of optical flow magnitude is greater than the empirical threshold value $V_{T}$. Both $O_{T}$ and $V_{T}$ should be adapted to the actual environments, and automatically calibrating the threshold value is left for future work, as mentioned in Section 6.

## 5. Experiments and Demonstrations

### 5.1. Experimental Setup

**Figure 8:**
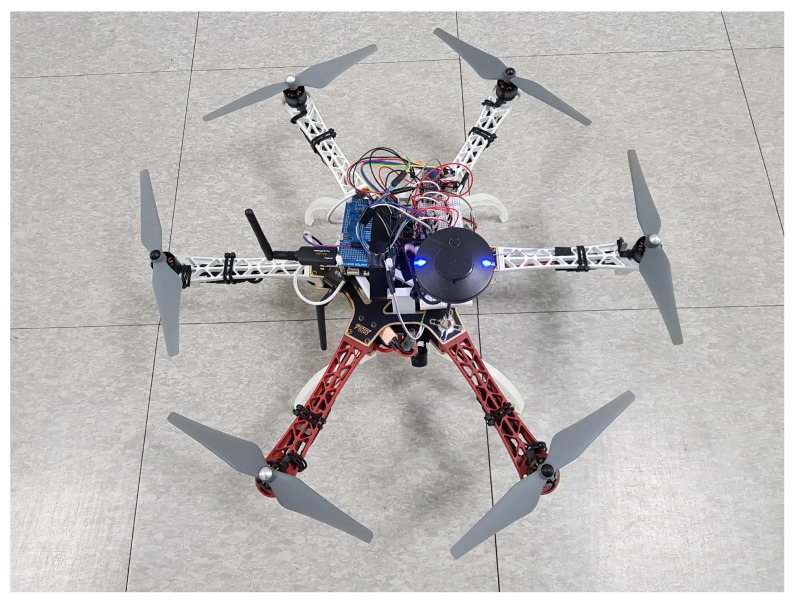
Prototype implementation of the system.

The implementation of the proposed system is based on our previous work [42,43]. For flight guidance system, twelve Cadmium Sulfide (CDS) light sensors were attached to the UAV in different directions. Also, a camera for the safe landing system was mounted on the UAV. Figure 8 shows a prototype of the UAV for proposed system. We adopted DJI’s F550 ARF KIT for the frame and HardKernel’s ODROID XU4 for the processing unit. The processing unit is connected to the light sensors, a camera, and a communication interface. In experiments, the camera was ODROID USB-CAM 720P, which has 720 p resolution, 30 fps with color scale, and $\theta_{\max}$ was measured experimentally at about 21${}^{\circ }$.

### 5.2. Flight Guidance System Demonstration

Prior to the guidance experiment, we confirmed that the light sensor can detect the laser in various environments. We measured the brightness of the light under a sunny, cloudy, night, and indoor with fluorescent light while the light sensor and the lasers were 15 m and 30 m away. For this experiment, we used a commercial laser. Table 2 shows the average of the measured brightness values. This experiment shows that laser can be distinguished under any environments. Even in the sunny, the brightest environment of all environments, the laser detector was able to identify the laser. Through this experiment, we were able to determine the threshold value to discriminate the laser. We also set the number of particles to 500, as mentioned in Section 3.2.4, and set the threshold ($N_{T}$) for the sample dispersion to 250, which is half the number of particles.

**Figure 9:**
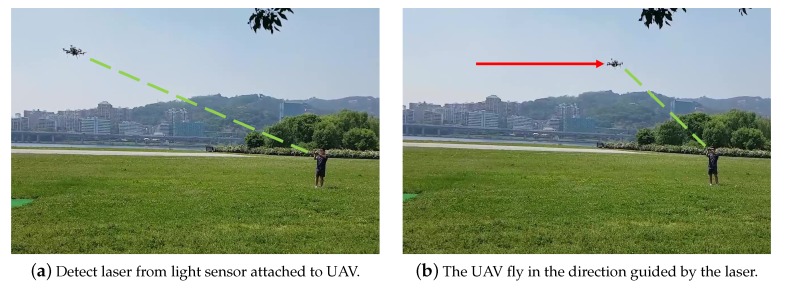
Laser guidance demonstration.

We used the UAV shown in Figure 8 to confirm that the flight guidance system is working properly. As shown in Figure 9a, the laser was aimed at the UAV in the autonomous flight. In addition, as shown in Figure 9b, we confirmed that the UAV was flying in the guided direction. The demonstration video can be seen on the following link [15].

In an additional experiment, we measured the time lag from the moment the laser was emitted toward the UAV to estimate the coordinates to move along that direction. In particular, the time lag includes resampling and direction estimation as well as updating the state and weight of each particle. We also calculated the RMSE of the difference between the first estimated flight direction and the constant measurements right after the initialization phase, and Table 3 shows the results depending on the number of particles. In Table 3, the average time lag of the currently implemented system with 500 particles was 91.7 milliseconds. The impact of this result is expected to be negligible when the UAV with a loss of GPS signal is hovering in place. Furthermore, as the number of particles decreased, the time lag was reduced, while the accuracy of the flight direction was significantly compromised. On the contrary, as the number of particles increased, the accuracy was improved, but the time was delayed too much. As mentioned in Section 3.2.4, it is very important to select and implement the optimal number of particles for each system.

### 5.3. Safe Landing System Demonstration

The safe landing system proved its performance through UAV landing experiments. In this experiment, when the UAV was lowering its altitude for landing, an optical flow magnitude map was generated from the image taken by the downward-facing camera. At this time, we confirmed whether the safe landing system can detect obstacles based on the optical flow magnitude map and whether the UAV can move in the direction of avoiding obstacles.

**Figure 10:**
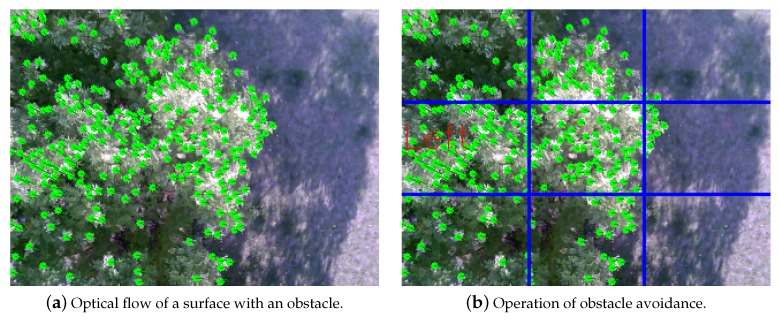
Optical flow magnitude map of obstacle avoidance.

Figure 10 represents the optical flow magnitude map of the image viewed by the camera facing downward of the UAV. In Figure 10a, the green dot represents the feature point, and the green line represents the optical flow calculated at the feature point. As the goodFeaturesToTrack function detects the strong corner and tends to detect from obstacles that are visually different from the floor and higher in height, Figure 10 shows the feature points mainly presented on the obstacle. As shown in Figure 10a, the left side of the tree is the highest obstacle, and the UAV obtains the highest optical flow magnitude values from the tree on the left side. As shown in Figure 10b, the OpenCV on the UAV recognized that the nearest obstacle was on the left. In this experiment, the safe landing system successfully maneuvered the UAV to avoid obstacles. Also, the frame rate was 31.1 FPS which is about five times faster than the previous work [14], 5.9 FPS which calculates the optical flow for all pixels.

**Figure 11:**
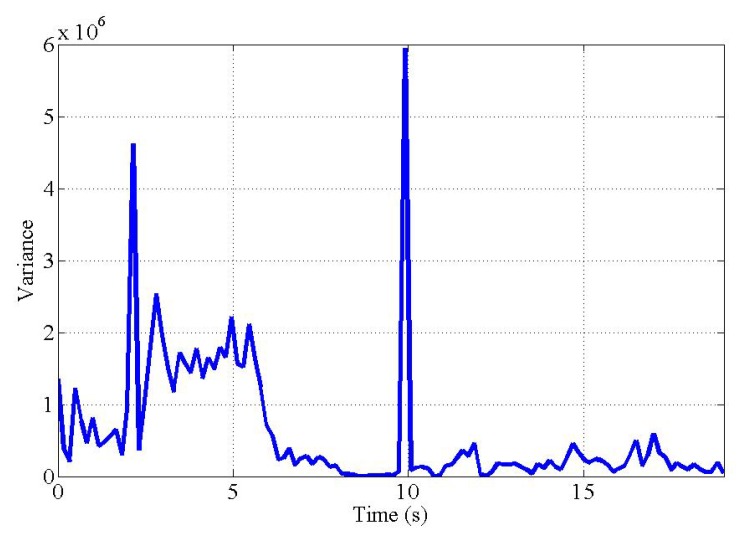
Variance of optical flow magnitude.

The variance measured during the experiment also shows that the UAV was able to recognize obstacles successfully. Figure 11 shows the variance of the optical flow magnitude in each segmented screen during the experiment. During obstacle avoidance, obstacles are detected in the left segmented screen, resulting in high optical flow magnitude in the left segmented screen. Therefore, the variance value of segmented screens was high until 10 s when the UAV was avoiding the obstacles. After the UAV completely avoided the obstacle, the sharp increase in variance at 10 s is shown in Figure 11. This is a temporary phenomenon that occurs while creating a new feature point on the ground because there are no more obstacles. After creating the feature points of the ground, the variance of the optical flow magnitude was measured low because the optical flow magnitude is evenly measured on each segmented screen. The full demonstration can be seen in the following link [16].

### 5.4. UAV Detour System Demonstration

Finally, we demonstrated the UAV detour system that consists of the flight guidance system and safe landing system. In this demonstration, the operator used a laser to guide the UAV to the landing point where the operator stood. The UAV’s flight guidance system identified the laser, guided the UAV to the landing point, and then proceeded to land. Since the operator was standing at the landing point of the UAV, the safe landing system recognized the operator as an obstacle, then automatically avoided the operator and landed safely at that point. The demonstration video of the UAV detour system can be seen on the following link [17].

## 6. Future Work

The flight guidance system can detect laser, but cannot identify malicious lasers that are intended to interfere with UAV movement. To solve this problem, as future work, we will develop a paring system so that only certified lasers can take control of the UAV. We plan to improve the system through a bandpass filter so that the sensor can identify lasers with a specific wavelength. If the data bit is transmitted through a laser, an encryption technique can be applied to the laser. This improvement will allow the UAV to identify the laser containing the certified data bit and move in that direction. Also, in an environment where a line of sight (LoS) is not secured, it is difficult to guide UAV with a laser. To cope with this environment, we are developing a system that guides UAV with extra media that can be used even if LoS is not secured (e.g., ultrasound). In addition, The flight guidance system requires the operator to operate the laser. To solve this inconvenience, we will develop an improved landing point system that identifies UAVs through image processing and automatically aims the laser. Overall, we will improve the flight guidance system to suit the delivery system in an urban area.

The safe landing system will be extended to automatically avoid obstacles that UAVs can encounter during the entire process of takeoff, flying, and landing to perform their mission in an urban area. In addition, the values we set as the threshold (e.g., $O_{T}$, $V_{T}$) should be set in response to various circumstances. We are setting it as a future goal to make automatic calibration through machine learning.

## 7. Conclusions

UAVs can perform various missions. Some of UAVs performing missions are capturing video or collecting information over an extensive area, and some UAVs perform missions in urban areas such as delivery UAVs. However, in urban areas, buildings weaken the GPS signal, and there are many obstacles that disturb the UAVs’ flight. Therefore, UAV flying in urban area requires additional systems to fly in the absence of GPS or to avoid obstacles. This paper proposes the UAV detour system considering UAVs performing missions in urban areas. The UAV detour system allows the UAV to fly and land in situations where GPS or networks are disconnected. The flight guidance system, which is one of the subsystems of the UAV detour system, maneuvers the UAV by using a laser that is not disturbed by various radio waves or signal interference. Another subsystem, the safe landing system, identifies obstacles based on optical flow, allowing the UAV to avoid obstacles when landing. Finally, the proposed subsystems were tested on a prototype UAV. The performance of subsystems were verified by successfully performing flight guidance and obstacle avoidance landing.

## Figures and Tables

**Table 1:** Comparison of root mean square error (RMSE) performance depending on the number of particles.

Number of Particles	100	500	2000	10,000
**RMSE (rad)**	0.047	0.011	0.004	0.002
**RMSE (degree)**	2.712	0.609	0.204	0.129

**Table 2:** Measurement of light intensity in various environments.

	Distance (15 m)		Distance (30 m)	
	Without Laser (lx)	Laser Projected (lx)	Without Laser (lx)	Laser Projected (lx)
Sunny	10,820.45	30,306.2	11781.6	20,264.17
Cloudy	7031.69	31,509.4	7250.12	15,792
Night	2.31	32,870.2	2.82	14,827.4
Indoor (flourescent light)	202.59	41,238.2	133.4	24,834

**Table 3:** Comparison of performance depending on the number of particles in real experiment.

Number of Particles	100	500	2000	10,000
**Average time lag (ms)**	27.2	91.7	397.1	1642.4
**RMSE (rad)**	0.0568	0.0239	0.0118	0.0047

## References

[1] Park S., Lee J.Y., Um I., Joe C., Kim H.T., Kim H. (2019). RC Function Virtualization-You Can Remote Control Drone Squadrons. Proceedings of the 17th Annual International Conference on Mobile Systems, Applications, and Services.

[2] Hart W.S., Gharaibeh N.G. Use of micro unmanned aerial vehicles in roadside condition surveys. Proceedings of the Transportation and Development Institute Congress 2011: Integrated Transportation and Development for a Better Tomorrow.

[3] Cheng P., Zhou G., Zheng Z. Detecting and counting vehicles from small low-cost UAV images. Proceedings of the ASPRS 2009 Annual Conference.

[4] Jensen O.B. (2016). Drone city—Power, design and aerial mobility in the age of “smart cities”. Geogr. Helv..

[5] Jung J., Yoo S., La W., Lee D., Bae M., Kim H. (2018). Avss: Airborne video surveillance system. Sensors.

[6] Bae M., Yoo S., Jung J., Park S., Kim K., Lee J., Kim H. (2018). Devising Mobile Sensing and Actuation Infrastructure with Drones. Sensors.

[7] Chung A.Y., Jung J., Kim K., Lee H.K., Lee J., Lee S.K., Yoo S., Kim H. (2015). Poster: Swarming drones can connect you to the network. Proceedings of the 13th Annual International Conference on Mobile Systems, Applications, and Services, MobiSys 2015.

[8] Park S., Kim K., Kim H., Kim H. (2018). Formation control algorithm of multi-UAV-based network infrastructure. Appl. Sci..

[9] Farris E., William F.M.I. (2016). System and Method for Controlling Drone Delivery or Pick Up during a Delivery or Pick Up Phase of Drone Operation. U.S. Patent.

[10] Kimchi G., Buchmueller D., Green S.A., Beckman B.C., Isaacs S., Navot A., Hensel F., Bar-Zeev A., Rault S.S.J.M. (2014). Unmanned Aerial Vehicle Delivery System. U.S. Patent.

[11] Paek J., Kim J., Govindan R. (2010). Energy-efficient Rate-adaptive GPS-based Positioning for Smartphones. Proceedings of the 8th International Conference on Mobile Systems, Applications, and Services.

[12] QUARTZ Amazon Drones won’t Replace the Mailman or FedEx Woman any Time soon. http://qz.com/152596.

[13] Nguyen P., Ravindranatha M., Nguyen A., Han R., Vu T. (2016). Investigating cost-effective rf-based detection of drones. Proceedings of the 2nd Workshop on Micro Aerial Vehicle Networks, Systems, and Applications for Civilian Use.

[14] Chung A.Y., Lee J.Y., Kim H. Autonomous mission completion system for disconnected delivery drones in urban area. Proceedings of the 2017 IEEE International Conference on Robotics and Biomimetics (ROBIO).

[15] Lee J.Y., Shim H., Park S., Kim H. Flight Path Guidance System. https://youtu.be/vjX0nKODgqU.

[16] Lee J.Y., Joe C., Park S., Kim H. Safe Landing System. https://youtu.be/VSHTZG1XVLs.

[17] Lee J.Y., Shim H., Joe C., Park S., Kim H. UAV Detour System. https://youtu.be/IQn9M1OHXCQ.

[18] Stary V., Krivanek V., Stefek A. (2018). Optical detection methods for laser guided unmanned devices. J. Commun. Netw..

[19] Shaqura M., Alzuhair K., Abdellatif F., Shamma J.S. Human Supervised Multirotor UAV System Design for Inspection Applications. Proceedings of the 2018 IEEE International Symposium on Safety, Security, and Rescue Robotics (SSRR).

[20] Jang W., Miwa M., Shim J., Young M. (2017). Location Holding System of Quad Rotor Unmanned Aerial Vehicle (UAV) using Laser Guide Beam. Int. J. Appl. Eng. Res..

[21] Fox D., Burgard W., Kruppa H., Thrun S. (2000). A probabilistic approach to collaborative multi-robot localization. Auton. Robot..

[22] Hightower J., Borriello G. (2004). Particle filters for location estimation in ubiquitous computing: A case study. Proceedings of the International conference on ubiquitous computing.

[23] Rosa L., Hamel T., Mahony R., Samson C. (2014). Optical-flow based strategies for landing vtol uavs in cluttered environments. IFAC Proc. Vol..

[24] Souhila K., Karim A. (2007). Optical flow based robot obstacle avoidance. Int. J. Adv. Robot. Syst..

[25] Yoo D.W., Won D.Y., Tahk M.J. (2011). Optical flow based collision avoidance of multi-rotor uavs in urban environments. Int. J. Aeronaut. Space Sci..

[26] Miller A., Miller B., Popov A., Stepanyan K. (2019). UAV Landing Based on the Optical Flow Videonavigation. Sensors.

[27] Herissé B., Hamel T., Mahony R., Russotto F.X. (2011). Landing a VTOL unmanned aerial vehicle on a moving platform using optical flow. IEEE Trans. Robot..

[28] Mori T., Scherer S. First results in detecting and avoiding frontal obstacles from a monocular camera for micro unmanned aerial vehicles. Proceedings of the 2013 IEEE International Conference on Robotics and Automation.

[29] Hrabar S. 3D path planning and stereo-based obstacle avoidance for rotorcraft UAVs. Proceedings of the 2008 IEEE/RSJ International Conference on Intelligent Robots and Systems.

[30] Ferrick A., Fish J., Venator E., Lee G.S. UAV obstacle avoidance using image processing techniques. Proceedings of the 2012 IEEE International Conference on Technologies for Practical Robot Applications (TePRA).

[31] Kendoul F. (2012). Survey of advances in guidance, navigation, and control of unmanned rotorcraft systems. J. Field Robot..

[32] Bi Y., Duan H. (2013). Implementation of autonomous visual tracking and landing for a low-cost quadrotor. Opt.-Int. J. Light Electron Opt..

[33] Lange S., Sünderhauf N., Protzel P. A vision based onboard approach for landing and position control of an autonomous multirotor UAV in GPS-denied environments. Proceedings of the 2009 International Conference on Advanced Robotics.

[34] Venugopalan T., Taher T., Barbastathis G. Autonomous landing of an Unmanned Aerial Vehicle on an autonomous marine vehicle. Proceedings of the 2012 Oceans.

[35] Cesetti A., Frontoni E., Mancini A., Zingaretti P., Longhi S. (2010). A vision-based guidance system for UAV navigation and safe landing using natural landmarks. Proceedings of the Selected papers from the 2nd International Symposium on UAVs.

[36] Eendebak P., van Eekeren A., den Hollander R. (2013). Landing spot selection for UAV emergency landing. Proceedings of the SPIE Defense, Security, and Sensing.

[37] Ristic B., Arulampalam S., Gordon N. (2004). Beyond the Kalman filter. IEEE Aerosp. Electron. Syst. Mag..

[38] Lucas B.D., Kanade T. An iterative image registration technique with an application to stereo vision. Proceedings of the 7th International Joint Conference (IJCAI) 1981.

[39] Farnebäck G. (2003). Two-frame motion estimation based on polynomial expansion. Proceedings of the Scandinavian Conference on Image Analysis.

[40] Bouguet J.Y. (2001). Pyramidal implementation of the affine lucas kanade feature tracker description of the algorithm. Intel Corp..

[41] Bradski G. (2000). The opencv library. Dr Dobb’s J. Software Tools.

[42] Yoo S., Kim K., Jung J., Chung A.Y., Lee J., Lee S.K., Lee H.K., Kim H. A Multi-Drone Platform for Empowering Drones’ Teamwork. http://youtu.be/lFaWsEmiQvw.

[43] Chung A.Y., Jung J., Kim K., Lee H.K., Lee J., Lee S.K., Yoo S., Kim H. Swarming Drones Can Connect You to the Network. https://youtu.be/zqRQ9W-76oM.

